# Annual Research Review: Towards a deeper understanding of nature and nurture: combining family‐based quasi‐experimental methods with genomic data

**DOI:** 10.1111/jcpp.13720

**Published:** 2022-11-15

**Authors:** Tom A. McAdams, Rosa Cheesman, Yasmin I. Ahmadzadeh

**Affiliations:** ^1^ Social Genetic and Developmental Psychiatry Centre, Institute of Psychiatry, Psychology and Neuroscience King's College London London UK; ^2^ PROMENTA Research Centre, Department of Psychology University of Oslo Oslo Norway

**Keywords:** Quasi‐experiment, adoption, sibling comparison, children‐of‐twins, extended family, intergenerational, gene–environment correlation, causal inference, direct and indirect genetic effects, genetic nurture, dynastic effects

## Abstract

Distinguishing between the effects of nature and nurture constitutes a major research goal for those interested in understanding human development. It is known, for example, that many parent traits predict mental health outcomes in children, but the causal processes underlying such associations are often unclear. Family‐based quasi‐experimental designs such as sibling comparison, adoption and extended family studies have been used for decades to distinguish the genetic transmission of risk from the environmental effects family members potentially have on one another. Recently, these designs have been combined with genomic data, and this combination is fuelling a range of exciting methodological advances. In this review we explore these advances – highlighting the ways in which they have been applied to date and considering what they are likely to teach us in the coming years about the aetiology and intergenerational transmission of psychopathology.

## Introduction

A serious challenge to any attempt at understanding the forces that shape human development is the non‐independence of nature and nurture (Plomin, DeFries, & Loehlin, 1977; Scarr & McCartney, 1983). Parents are the source of both DNA and a rearing environment, and this makes it difficult to distinguish the effects of one from the other. For example, parent psychopathology predicts offspring psychopathology. This could be because parent mental health problems are detrimental to offspring well‐being and/or because children inherit genetic risk from their parents. Similarly, estimates of genetic influence on mental health may capture not only the direct genetic effects of an individual's DNA, but also the effects of relatives, whose (partially shared) genomes contribute to the rearing environment (Bates et al., 2018; Kong et al., 2018). Correlation between genetic and environmental influences means we cannot know the influence of parent behaviour on child development without controlling for the DNA that parent and child share. Likewise, we cannot know the influence of an individual's DNA without controlling for the DNA of their family members.

Researchers interested in parental effects have employed a range of family‐based quasi‐experimental methods to evaluate the influence of parent on child after accounting for the confounding effects of shared genes and environments. These methods include sibling comparison, adoption studies and intergenerational extended family studies (D'Onofrio, Lahey, Turkheimer, & Lichtenstein, 2013). The genius of these designs is that they allow researchers to control for genetic and environmental confounds without the need to identify or directly measure them. Over the last two decades, family‐based quasi‐experimental methods have played a key role in advancing our understanding of the intergenerational transmission of psychopathology (see systematic reviews by Jami, Hammerschlag, Bartels, & Middeldorp, 2021; McAdams et al., 2014).

In recent years, genomic data have been collected on many of the participants of family data sets. Large biobanks have also been created that (by chance and by design) contain genotyped relatives. These new data are giving rise to exciting new research possibilities, whereby DNA is being combined with family‐based quasi‐experimental methods. This combination provides researchers with new tools to disentangle the effects of nature and nurture. For example, in the same way that family‐based quasi‐experimental methods provide the opportunity to control for unmeasured genetic confounding when studying family environmental effects, they can also be used to control for unmeasured environmental confounding when studying genetic effects (Bates et al., 2018; Brumpton et al., 2020; Eaves, Pourcain, Smith, York, & Evans, 2014; Howe et al., 2021; Kong et al., 2018; Morris, Davies, Hemani, & Smith, 2020; Visscher et al., 2006; Young et al., 2018).

**Figure 1:**
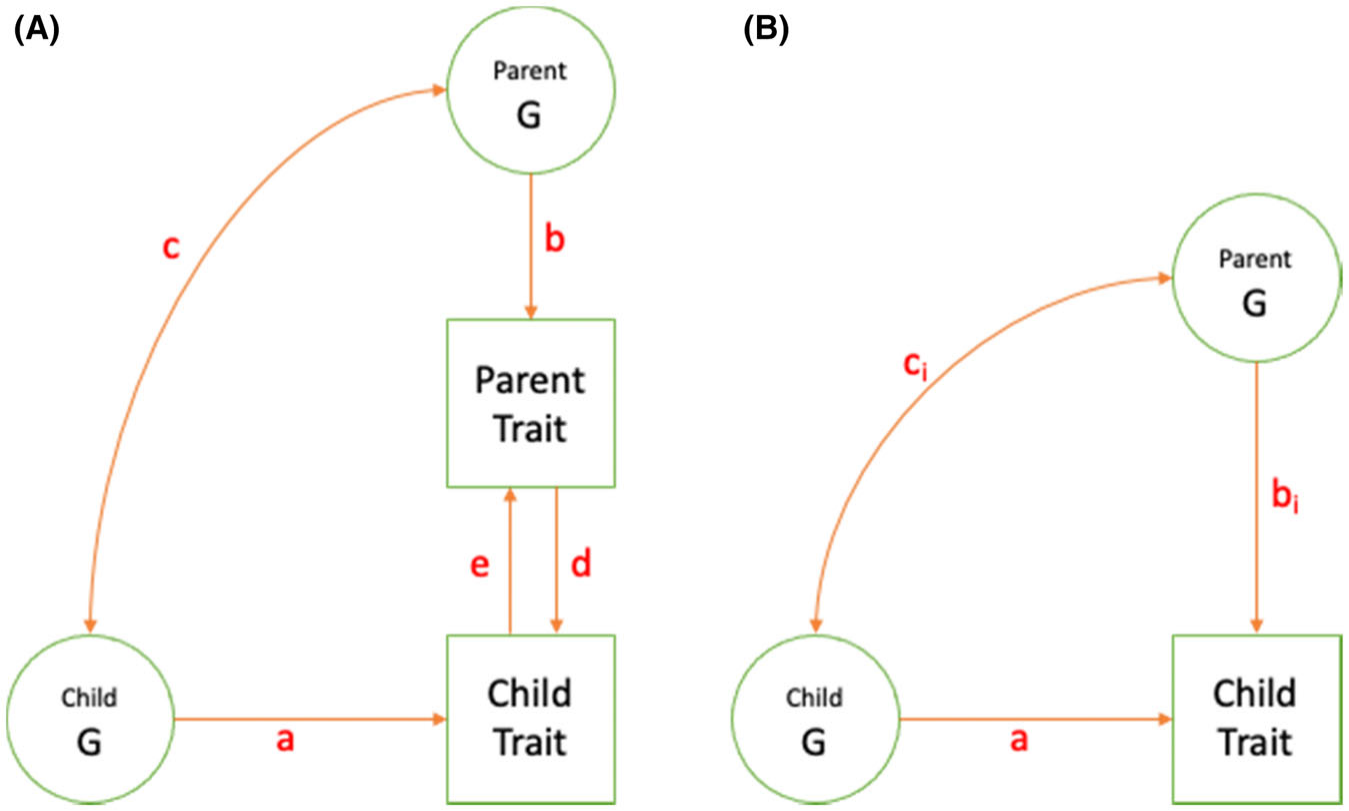
(A) Path diagram illustrating influence of parent trait and child trait on one another, and genetic covariance. a: causal influence of child genotype on child trait (*direct* genetic effect). b: causal influence of parent genotype on parent trait (*direct* genetic effect). c: correlation between child genotypic influences on child trait and parent genotypic influences on parent trait. d: causal influence of parent trait on child trait. e: causal influence of child trait on parent trait. (B) Path diagram illustrating influence of parent and child genomes on child trait and their covariance. a: causal influence of child genotype on child trait (*direct* genetic effect). b_i_: causal influence of parent genotype on child trait (*indirect* genetic effect of parent on child trait: genetic nurture). c_i_: correlation between child genotypic and parent genotypic influences on child trait. Note that residual variances and non‐genetic influences and confounders are not included. Parent and child genotypes may be latent (e.g. variance components) or manifest (e.g. polygenic scores or specific variants). Assortative mating is not explicitly modelled in this path diagram (see glossary and discussion for details on the consequences of assortative mating)

In this review, we explore how some family‐based quasi‐experimental methods have been adapted for the postgenomic era. The methods we review are largely focussed on tackling the non‐independence of nature (genetics) and nurture (the rearing environment) and what this means for our understanding of aetiology and intergenerational transmission. In reviewing this literature, we refer to several key concepts relevant to understanding this non‐independence. Some have been around for many years (gene–environment correlation), some are relatively new (genetic nurture), and several are overlapping (gene–environment correlation, indirect genetic effects, genetic nurture and dynastic effects). In Table 1 and Figure 1, we define these key concepts and clarify how they relate to one another. We also include an online glossary (Appendix S1) defining other quantitative genetic terms used in this review. We flag the first use of terms included in the glossary with an asterisk.

**Table 1:** Key concepts for understanding the non‐independence of nature and nurture

***Passive Gene–Environment Correlation* .** The passive inheritance of genotype and correlated environment (Plomin et al., 1977; Scarr & McCartney, 1983). Passive gene–environment correlation often refers to associations between child genome and the rearing environment, where parent genome influences both. For example, a parent's genotype may influence the hostility they show towards their child. Their child will inherit some of the genes associated with hostility from their parent. In this manner, the child's genome will correlate with the hostility they are exposed to. In Figure 1A, passive gene–environment correlation is evident if paths b and c are non‐zero. Child genes that correlate with their environment are likely to influence child phenotype as well (path a). For example, genes that influence hostility in a parent may, when passed to their child, influence conduct problems. In such a scenario, estimates of the effects of parent hostility on child conduct problems may be confounded by passive gene–environment correlation (also sometimes referred to as genetic confounding). In Figure 1A, if paths a, b and c are non‐zero, then the correlation between parent and child trait will be (at least partially) attributable to passive gene–environment correlation. To accurately estimate path d (causal effect of parent trait on child trait), it is necessary to control for path a‐ > c‐ > b (confounding by passive gene–environment correlation).
** *Evocative Gene–Environment Correlation* .** A correlation between genotype and environment, arising when an individuals' genotype evokes a change in their environment (Plomin et al., 1977; Scarr & McCartney, 1983). For example, if a child's genetically influenced conduct problems evoked hostile behaviour from their parents, this would induce a correlation between child genome and parent hostility. In Figure 1A, evocative gene–environment correlation would be evident if paths a and e were non‐zero, thereby (at least partially) explaining the association between child genome and parent trait.
** *Direct Genetic Effects* .** The direct causal effects of an individual's genotype on their phenotype, independent of phenomena that lead to non‐causal associations between genotype and phenotype (Kong et al., 2018; Lynch & Walsh, 1998). In Figure 1, path a indexes the direct genetic effects of the child. (Path b indexes direct genetic effects of the parent.) Estimates of direct genetic effects can be biased by various phenomena including passive gene–environment correlation and genetic nurture (see below).
** *Indirect Genetic Effects* .** The effect of one individual's genotype on another's phenotype via environmental pathways (Lynch & Walsh, 1998). Indirect genetic effects can originate from the genotypes of relatives or non‐relatives. For example, an adolescent's genome may indirectly influence the delinquent behaviour of their friends via social processes.
** *Genetic Nurture/Dynastic Effects* ** . Terms used to describe the indirect genetic effects of family members on one another (e.g. Kong et al., 2018; Morris et al., 2020). Genetic nurture is most often used to refer to the influence of parent genotype on child phenotype, via environmental pathways. For example, if parent genotype influences parent hostility, this may in turn influence child conduct problems. In Figure 1A, the chain of paths running b‐ > d index genetic nurture via a specific parent trait. In Figure 1B, path b_i_ indexes total genetic nurture associated with parent genotype, without reference to an intermediate parent phenotype. Genetic nurture is of interest to social scientists because it can index family environmental influences on a phenotype. It is also of interest to researchers concerned with estimating direct genetic effects because genetic nurture can bias estimates of direct genetic effects. For example, genes that influence child conduct problems indirectly via parent behaviour may also influence child conduct problems directly as part of the child genome. In such a scenario, estimates of the direct genetic effects of child genotype on child phenotype will be biased unless the indirect genetic effects of parent genotype (genetic nurture) are accounted for. In Figure 1A, to accurately estimate path a (direct genetic effects on child trait), it is necessary to control for the alternate path running d‐ > b‐ > c (Figure 1A) or b_i_‐ > c_i_ (Figure 1B) (each indexing potential confounding by genetic nurture).

### What can be achieved by combining genomic data with family‐based quasi‐experimental methods?

The combination of genomic data and family‐based quasi‐experimental methods is providing researchers with new tools to use in assessing the impact of parents on children, and children on parents. They are also providing fresh insight into genomic prediction. While it is widely appreciated that associations between parent and child phenotypes can be confounded by their genetic relatedness, it is perhaps less widely appreciated that associations between a person's genotype and their phenotype can also be confounded. Genetic nurture (Table 1), population stratification* and assortative mating* can bias associations between a person's genotype and their phenotype (Bates et al., 2018; Brumpton et al., 2020; Eaves et al., 2014; Howe et al., 2021; Kong et al., 2018; Morris et al., 2020; Visscher et al., 2006; Young et al., 2018). Understanding the potential for genotype–phenotype associations to be biased by such phenomena is important given the increasing use of genetic data by researchers studying human traits. For example, genome‐wide association studies* (GWAS) have implicated hundreds of genetic variants in the aetiology of mental health (Trubetskoy et al., 2022). However, identified variants can tell us little about trait biology if associations reflect influences besides direct causal effects. Polygenic scores* (PGS) are often interpreted as indexing individual genetic risk for a trait (i.e. *direct* genetic effects), but can also capture the environmental risk of their family (Kong et al., 2018). Importantly, family‐based quasi‐experimental research designs provide researchers with the opportunity to distinguish direct genetic effects from genetic nurture, population stratification and assortative mating.

In this review, we explore how three of the most popular family‐based quasi‐experimental designs – sibling comparison, adoption and intergenerational extended family studies – have been adapted for the postgenomic era. For each we first introduce the logic of the models as they have been traditionally applied to the genetically informed study of intergenerational associations (i.e. without the inclusion of DNA). We then describe how researchers have used these designs when genome‐wide genomic data have been available and what we have learnt about 1. the family environment and 2. genomic prediction in doing so. (In this review, we do not consider the use of single candidate genes.) We focus primarily on psychopathology, but where methods have not yet been applied to mental health, we discuss those phenotypes they have been applied to. We also look to the future and consider ways in which these methods will further our understanding of psychopathology over the coming years.

## Sibling comparison studies

Sibling comparison studies can be used to study associations between parent and child phenotypes while controlling for genetic and environmental confounds (Lahey & D'Onofrio, 2010; Petersen & Lange, 2020). Siblings are informative because they share parents, a family environment and genetic risk. As such, if siblings' differential exposure to a risk factor (e.g. maternal use of selective serotonin reuptake inhibitors during pregnancy) predicts differences in an outcome (e.g. childhood anxiety), then the association cannot be attributed to potential confounding effects shared between siblings (Brandlistuen et al., 2015). Where a parent–child association is attributable to confounding factors, then within‐sibship estimates based on sibling comparison will be different to population estimates, and this difference will indicate the magnitude of confounding^1^.

Importantly, the sibling‐comparison design can control for confounding by passive gene–environment correlation (the possibility that parent–child associations are attributable to genetic relatedness) even though siblings differ genetically. This is because the random assignment of alleles* at conception means that genetic differences between siblings do not correlate with differences in environmental exposures. This of course is assuming that sibling‐to‐sibling effects do not bias associations, and that genetic differences between siblings do not evoke differences in parental exposure. This may be a reasonable assumption for some parent exposures, (e.g. antenatal depression or smoking during pregnancy^2^) but not all. For example, genetic differences between siblings may cause differences in child behaviour which could explain differences in parenting behaviours directed towards each child. In the language of gene–environment correlation (Table 1), sibling comparison studies control for passive but not evocative gene–environment correlation.

Sibling‐comparison studies have been used extensively in psychopathology research. Demonstrating, for example, that although exposure to maternal anxiety and depression during pregnancy predicts childhood emotional and behavioural problems in offspring, this association appears attributable to confounding influences shared by siblings, as differences in exposure between siblings do not predict differences in outcome (Bekkhus et al., 2018; Gjerde et al., 2017, 2020). Differences in *postnatal* exposure to maternal anxiety and depression on the other hand *do* predict differences in outcomes between siblings, suggesting that postnatal maternal mental health may have a causal impact on child well‐being (Gjerde et al., 2017, 2020; Kendler, Ohlsson, Sundquist, & Sundquist, 2020c).

## Genomic sibling comparison studies

### Using DNA in sibling comparison studies to improve our understanding of the family environment

As described above, sibling comparison studies can control for passive gene–environment correlation but cannot account for evocative gene–environment correlation. In many instances, this means that sibling‐comparison studies cannot tell us whether differences in exposure cause differences in outcome and/or whether differences between siblings cause differences in outcome. However, when DNA is collected from sibling pairs, within‐sibship genomic prediction can be used to explore evocative gene–environment effects. For example, dizygotic (DZ) twin differences for child BMI PGS have been shown to predict differences in the feeding behaviours parents direct towards twins (Selzam et al., 2018). This is an example of evocative gene–environment correlation, whereby the child genome has an indirect genetic effect on parent behaviour. The use of a PGS for BMI, as opposed to a measure of BMI itself, means that the direction of effects is clear – parent feeding behaviours cannot cause changes to child DNA, so the reverse must be true. This highlights one of the advantages of using DNA: if researchers examined associations between measured BMI and parenting behaviours – even within a sibling‐comparison design – it would be unclear whether BMI influences parent feeding behaviour and/or vice versa.

### Using sibling comparison to improve our understanding of genomic prediction

Where DNA is collected on samples of sibling pairs, genetic differences between siblings can be compared with phenotypic differences in much the same way that differences in exposure are compared with differences in outcome in traditional sibling comparison studies (Brumpton et al., 2020; Howe et al., 2021; Morris et al., 2020; Selzam et al., 2019; Visscher et al., 2006). For example, sibling differences in depression PGS can be used to predict differences in depression symptoms. Resultant within‐sibship estimates constitute the direct genetic effect of the PGS independent of environmental factors shared by siblings. This includes genetic nurture, population stratification and assortative mating (all non‐causal sources of genotype–phenotype association; see Table 1 and the glossary for definitions). If genotype–phenotype associations are biased by these influences, then within‐sibship estimates will be different to population estimates, and this difference will indicate the magnitude of bias. An example of this approach comes from a study of dizygotic twin pairs in which within‐sibship PGS predictions for educational outcomes and IQ were attenuated, suggesting a role for genetic nurture and/or assortative mating and population stratification. Attenuation of associations between other PGSs and phenotypes (height, BMI, health, neuroticism, ADHD symptoms, schizophrenia symptoms) was less pronounced and not significant (Selzam et al., 2019).

Sibling‐comparison GWAS require large samples of sibling pairs, so few have been conducted (Hemani et al., 2013; Visscher et al., 2006; Young et al., 2018). This is likely to change, however, as more biobanks (that often include many sibling pairs by chance) and family‐based studies are established. A recent example combined data from 17 studies to perform within‐sibship GWAS on 25 phenotypes in 68,691 sibships (Howe et al., 2021). Of these, within‐ sibship SNP effect sizes were significantly smaller than population estimates for six of 25 traits, most of which were behavioural in nature (age at first birth, educational attainment, depressive symptoms, smoking, cognitive ability and height). Within‐sibship SNP‐heritability* estimates for educational attainment, cognition and height were all substantially attenuated relative to population estimates, by 71%, 24% and 17% respectively, indicating that genetic nurture and/or population stratification and assortative mating explain a portion of the heritability of these traits. The same within‐sibship data also revealed important evidence that genetic correlations between some traits were attributable to population phenomena such as cross‐trait assortative mating, rather than a shared aetiology (a finding previously demonstrated using extended twin family data; Keller et al., 2013).

In summary, the combination of genomic data and sibling‐comparison methodology provides new means to explore gene–environment correlations. The ability to control for a wide range of unmeasured confounding when exploring genetic effects provides a powerful tool for researchers interested in distinguishing direct genetic effects from genetic nurture, population stratification and assortative mating (Hemani et al., 2013; Howe et al., 2021). Crucially, knowledge about the genetic differences between siblings also provides a route through which researchers can explore the impacts of children's heritable traits on parents (evocative gene–environment correlation), something not possible in traditional sibling‐comparison designs (Selzam et al., 2018).

## Adoption studies

Adoption studies provide another means of distinguishing nature from nurture. When adoptive parents are unrelated to their adopted children, associations between parent and child traits cannot be attributed to shared genetics, so provide insight into the effects of parents on children (and vice versa) without confounding by passive gene–environment correlation (Table 1) (Leve et al., 2013; Plomin & DeFries, 1985). When children are adopted at birth, associations between birth parent and child cannot be attributed to postnatal factors and so can be used as a proxy indicator for the effects of genetic and/or prenatal environmental factors. Adoption‐at‐conception studies focus on children conceived via gamete or embryo donation and provide a method for distinguishing the effects of the prenatal environment from inherited genetic factors (Rice et al., 2010; Rice, Langley, Woodford, Davey Smith, & Thapar, 2018; Thapar et al., 2007).

In psychopathology research, adoption studies have played a key role in demonstrating that maternal depression is predictive of emotional and behavioural problems in offspring even when mother and child are not genetically related (see Natsuaki et al. (2014) for a review covering many such studies). They have also shown that offspring psychopathology can predict parent mental health (Ahmadzadeh et al., 2019; Brooker et al., 2015; McAdams et al., 2015) and parenting practices (Natsuaki et al., 2013; Trentacosta et al., 2019) via environmental pathways.

## Genomic adoption studies

### Using DNA in adoption studies to improve our understanding of the family environment

Collecting DNA from adoptees and/or their parents provides researchers with new methods to explore the effects of parent on child, and vice versa. For example, by examining associations between parent PGS for educational attainment and the educational attainment of adopted children, researchers have demonstrated a ‘genetic nurture’ effect even where parent and child are not genetically related (Domingue & Fletcher, 2020). This suggests that the rearing environment provided by the parent (as indexed by their PGS for educational attainment) is influencing child educational attainment. Of course, in a classical adoption study (that does not include DNA), it is also possible to assess the impact of adoptive parents on their adopted children and thus gain insight into the effects of parents on children independent of genetic relatedness. An advantage of using DNA to do this is that researchers can explore associations between *any* parent PGS and a child outcome. Without DNA researchers are confined to study only those phenotypes that have been directly assessed. Furthermore, in adoption studies associations between parent and child phenotypes can still be confounded even though passive gene–environment correlation is controlled for. For example, shared environmental influences could cause both parent and child mental health issues and thus explain their association. However, such an explanation would not explain associations between a parent's depression PGS and their adopted child's mental health. When DNA is collected on adopted *children*, the converse is true – researchers can evaluate associations between any child PGS and parent behaviour as a way of assessing the indirect genetic effects of children on parents (evocative rGE) independent of relatedness and environmental confounding.

### Using adoption studies to improve our understanding of genomic prediction

Collecting DNA on adopted children and/or their parents also provides opportunities to establish genetic influence on a trait independent of the influence of being reared by biological parents. In samples of adoptees, genetic associations are not inflated by genetic nurture from relatives and thus provide a better index of direct genetic effects (Table 1) than can non‐adoptee samples. Where estimates of direct genetic effects are lower in samples of adoptees than non‐adoptees, then the difference between the two provides an indication of the extent of genetic nurture. For example, in the UK Biobank, it has been shown that the SNP heritability of educational attainment is lower in adoptees than non‐adoptees, and years‐of‐education PGS predicts only around half the variance (Cheesman et al., 2020). This reduced prediction implies that typical estimates of genetic associations involving educational attainment index not only the direct genetic effects of a person's DNA but also the environmental effects of living with relatives (genetic nurture). However, this may not be the case for all traits: in an analysis using the same database, it was found that SNP heritability and PGS prediction of a range of body composition traits (BMI, body fat, fat mass, fat‐free mass and waist‐to‐hip ratio) did not differ between adoptees and non‐adoptees (Hunjan et al., 2021).

There are some caveats to the use of adoption data to distinguish direct genetic effects and genetic nurture: First, it is possible for genetic nurture effects to occur prior to adoption. For example, perinatal genetic nurture effects from the birth mother to child could inflate estimates of direct genetic estimates in adoptees where they are assumed to include no such effects. Second, some adoptees may have been reared by relatives (e.g. grandparents). If so, weakened postnatal genetic nurturing effects could also inflate direct genetic estimates. Where data sets contain information on the timing of adoption and the identity of the adoptive parents, it will be possible to distinguish direct genetic effects from pre‐ and postnatal genetic nurture (Hwang, Moen, & Evans, 2021). Third, findings from adoption studies are limited in generalisability: Adoptive parents tend to be more educated, wealthier and display less psychopathology than other parents (Natsuaki et al., 2019). Adopted children are also at higher risk of prenatal adversity and inheriting genetic risk for psychopathology (Marceau et al., 2016). The experience of being raised by parents to whom they are not genetically related may also influence child development (Cadoret, 1990; Daniels & Thorn, 2001). An advantage of collecting genomic data on adoptees is that it can be used to explore issues relating to generalisability. For example, Cheesman, Hunjan, et al. (2020) demonstrated that although adoption status was itself heritable and correlated genetically with several other traits, heritability (and thus variance explained in other traits) was low. They also reported that genetic influences on education were largely the same in adoptees and non‐adoptees.

In summary, combining the methods of adoption studies with genomic data provides new ways to explore both the effects of the family environment and genetic influence. In the future, we anticipate that genomic adoption studies will help to further unravel the role of the family in the development of psychopathology. Initially, data to do this will likely come from biobanks and other data sets that happen to contain large numbers of identifiable adoptive families (e.g. Cheesman, Hunjan, et al., 2020; Domingue & Fletcher, 2020; Hunjan et al., 2021; Hwang et al., 2021). Studies focussed specifically on samples of adoptees and their adoptive families tend to comprise only a few hundred families, so have less statistical power than data sets derived from biobanks (Leve, Neiderhiser, Natsuaki, Shaw, & Reiss, 2019). However, as GWAS become larger, and PGSs explain more variance, PGS analyses in such samples will prove feasible. Should adoption studies collect DNA from parents *and* children, this will provide opportunities to simultaneously explore the effects of parent genome on child outcome and vice versa, all unconfounded by genetic relatedness.

## Intergenerational extended twin/family studies

Intergenerational extended twin/family studies can be used to estimate the extent to which risk for psychopathology is transmitted from parents to children via genetic versus environmental mechanisms. Such studies involve samples of parents and children embedded within extended families; for example, pairs of twins and their offspring, or pairs of siblings or cousins and their offspring. Within these samples, families that differ in how related individual members are to one another (e.g. monozygotic twins and their children versus dizygotic twins and their children) can be compared with one another. Differences in covariances between these families can then be used to estimate the extent to which associations between parent and child traits are attributable to genetic versus environmental transmission (D'Onofrio et al., 2003; Keller et al., 2009; Kuja‐Halkola, D'Onofrio, Larsson, & Lichtenstein, 2014; Maes et al., 2006; McAdams et al., 2018; Silberg & Eaves, 2004). Such data have been used to explore a wide range of associations, indicating for example, that although anxiety in adult parents predicts childhood anxiety in offspring, these traits share little to no genetic overlap (Ask et al., 2021; Eley et al., 2015). In contrast, adult and child ADHD share a common genetic aetiology, which explains most of the intergenerational association (Wechsler et al., 2022).

Various extensions of extended twin/family models are possible and have been used to evaluate the direction of effects between parenting traits and child psychopathology (Ahmadzadeh et al., 2022; Narusyte et al., 2008), assortative mating (through the inclusion of spouses and in‐laws; Keller et al., 2009, 2013; Torvik et al., 2020) and genetically mediated sensitivity to parent characteristics.

## Intergenerational genomic studies

A shortcoming of intergenerational extended twin/family studies is the reliance on large, difficult‐to‐create databases comprising extended pedigrees. Indeed, it can be argued that this is a shortcoming of all the methods we have described in this review – it can be challenging to recruit large numbers of complete sibling pairs, adoptive families or extended twin families into scientific studies. This limits the utility of methods that rely on such data. An alternative to using extended family pedigrees to study genetic versus environmental mechanisms underlying the intergenerational transmission of risk is to collect DNA from both generations in samples of parents and children. It can be easier to recruit parent–child dyads or trios into studies than it is extended families (or sibling pairs or adoptive families). In recent years, increasing numbers of studies have collected DNA from parent and child generations, and we would suggest that intergenerational genomic databases are likely to prove the easiest to create and access of all of those we discuss in this review. Like genomic sibling‐comparison studies and genomic adoption studies, genomic intergenerational studies can provide researchers with insight into both, family environmental effects and genomic prediction.

### Using DNA in intergenerational studies to improve our understanding of the family environment

Several recently developed methods to estimating SNP heritability have been proposed that distinguish parent indirect genetic effects from child direct genetic effects using DNA from mother–child dyads (Eaves et al., 2014; Horikoshi et al., 2016; Qiao et al., 2020; Srivastava et al., 2020; Warrington et al., 2018) or mother–father‐child trios (Eilertsen et al., 2021; Young et al., 2018). An application of these methods to psychopathology used data from the Norwegian Mother, Father and Child Birth Cohort (MoBa), revealing genetic nurture effects for child depression but not anxiety at age 8. Substantial direct genetic effects on child anxiety and depression were also found, after adjusting for parental genetic nurture (Cheesman et al., 2020; Jami et al., 2020).

Other uses of parent–child genomic data have focussed on PGS prediction. For example, researchers have shown that when jointly modelling the influence of maternal, paternal and child PGSs on child ADHD, child genetic effects are the greater, but maternal PGSs for ASD and neuroticism also predict child ADHD, suggesting some genetic nurture (Pingault et al., 2021)^3^. As an alternative to jointly modelling parent and child PGSs, researchers have proposed the calculation of PGSs using only those parent alleles not transmitted to their child (Bates et al., 2018; Kong et al., 2018). Where non‐transmitted parental alleles predict variance in a child outcome, this can be interpreted as evidence for genetic nurture.

As well as examining associations between parent genotype and child phenotype (Figure 1B), intergenerational genomic data can be used to explore which parent phenotypes impact child development, and vice versa (Figure 1A). For example, researchers have included parent phenotypes as covariates when estimating the effects of parent genotype on offspring phenotype, with attenuation of genetic nurture estimates being taken to indicate that effects operate via (or are at least captured by) the parent phenotype controlled for (Bates et al., 2018; Cheesman, Eilertsen, et al., 2020). One study applied structural equation modelling to explore associations between child PGS, parent PGS, child educational attainment and parenting behaviours, thereby exploring passive and evocative gene–environment correlation and genetic nurture simultaneously (Wertz et al., 2020). Recently it was shown that the distinction between transmitted and non‐transmitted alleles, when combined with structural equation models (originally conceived for use with extended family data), can be used to determine causal influences of parent trait on child trait (Balbona, Kim, & Keller, 2021; Kim, Balbona, & Keller, 2020). In the proposed model, the non‐transmitted parental PGS is used as an instrumental variable (a variable that influences the outcome only via the predictor and thus can be used to estimate the causal influence of predictor on outcome, as in Mendelian randomisation; Lawlor et al., 2017). The structural equation models used allow for the simultaneous estimation of genetic nurture, the causal influence of parent trait on child trait and assortative mating (Balbona et al., 2021; Kim et al., 2020).

### Using intergenerational data to improve our understanding of genomic prediction

The intergenerational genomic methods described above provide estimates of direct genetic effects independent of genetic nurture, population stratification and assortative mating. To date, most applications of these methods have suggested that the greater portion of most of SNP heritability estimates and PGS predictions are attributable to direct genetic effects (Bates et al., 2018; De Zeeuw et al., 2020; Kong et al., 2018). However, evidence also supports the idea that population estimates are often inflated. For example, several intergenerational genomic studies have demonstrated that the variance in educational attainment explained by educational attainment PGSs comprises both direct and indirect genetic effects, with PGS comprising non‐transmitted alleles (i.e. parent genetic influences) predicting 33–46% of the variance predicted by transmitted alleles (Bates et al., 2018; De Zeeuw et al., 2020; Kong et al., 2018). Applications of this approach to traits other than education have found less evidence for genetic nurture. For example, it has been reported that non‐transmitted alleles associated with BMI are not predictive of children being overweight (Schnurr et al., 2020). And non‐transmitted PGSs for educational attainment and ADHD have been reported as not predictive of ADHD symptoms in children (De Zeeuw et al., 2020).

In summary, the combination of intergenerational family data and PGSs is likely to prove a fruitful one in the future. Intergenerational genetic data sets are becoming more commonplace, and a range of methods are being developed that can make use of such data to better understand genomic prediction and the environmental effects of family members on one another. These methods are new so have not yet been applied extensively. However, we anticipate that they will contribute considerably to our understanding of mental health aetiology over the coming years.

## Discussion

In this review, we have explored some of the methodological developments and findings that have come out of the recent increase in availability of genome‐wide family data. We have shown that many of these developments build on, and share commonalities with, research methods previously applied to control for genetic confounding when evaluating associations between parent and child traits. Where traditional methods focussed on assessing parental effects independent of passive gene–environment correlation, these new methods allow researchers to assess direct genetic effects independent of genetic nurture. They also provide new tools for researchers to use in assessing the impact of parents on children, and children on parents. Below we reflect on these developments and the influence they may have on the study of psychopathology over the coming years.

### What do estimates of ‘genetic nurture’ capture?

A key motivation for combining family and genomic data has been distinguishing direct genetic effects from environmental influences associated with living with biological parents. These influences are often interpreted as an indicator of the indirect genetic effects of parents on children (genetic nurture). Such indirect genetic effects are of interest because they can provide a broad indicator of the importance of parental influence in the development of a trait. However, most methods do not typically distinguish the indirect genetic effects of parents from that of population stratification, assortative mating and the indirect genetic effects of other relatives (siblings, etc.). Below we discuss each of these influences in turn, why they can be difficult to distinguish from parental indirect genetic effects and some approaches that can help to tease them apart.

Population stratification is a well‐established source of bias when estimating genetic effects (Cardon & Palmer, 2003). Every population is composed of many subpopulations. And individuals within each subpopulation are more closely related to one another than to people in other subpopulations. Even when attempts are made to methodologically control for population stratification (using databases comprising people with a similar ancestry and/or through principal components analysis), some subtle population stratification is likely to remain and can be difficult to account for (Morris et al., 2020). A major strength of family‐based quasi‐experimental methods is that they can control even for this subtle population stratification when estimating direct genetic effects. This is because close family members share a common ancestry (they come from the same subpopulation as one another). However, this also means that it can be difficult to know to what extent estimates of genetic nurture index parental indirect genetic effects versus population stratification. Databases comprising three or more generations of genomic data will allow for the distinction between historic population structure (captured by grandparental DNA) and the effects of the nuclear family (captured by parental DNA after accounting for grandparent effects).

Assortative mating can induce covariance between paternal and maternal genotypes associated with any trait assorted on. This means that estimates of genetic nurture involving such a trait (or correlated PGS) can be biased. For example, imagine estimating the indirect genetic effect of maternal depression PGS on child depression using a maternal PGS comprised only of non‐transmitted alleles. Assortative mating would induce covariance between the non‐transmitted alleles of the mother and the transmitted alleles of the father, meaning that estimates of genetic nurture would partially capture direct (as well as indirect) genetic effects (Balbona et al., 2021; Kim et al., 2020; Kong et al., 2018). This problem also biases estimates of indirect genetic effects of the maternal PGS on child trait when statistically controlling for child PGS for the same reason – the remaining prediction captures direct as well as indirect genetic effects. When DNA is available on both parents, the effects of assortative mating can be estimated using some of the methods discussed in this review (Balbona et al., 2021; Kim et al., 2020; Kong et al., 2018), as well as via triangulation across genomic adoption, sibling and parent–offspring designs, which are differentially affected by assortment (Demange et al., 2022). Where not explicitly modelled, assortative mating may bias estimates.

Besides population stratification and assortative mating, estimates of genetic nurture can capture the indirect genetic effects not only of parents but also siblings and other relatives. Where appropriate data are available, it is possible to distinguish maternal and paternal genetic influences (Eilertsen et al., 2021) and to estimate the effects of siblings (Kong et al., 2018) and grandparents (Liu, 2018). Identifying the source of genetic nurture effects and the mechanisms through which they operate is likely to be a research focus over the coming years. Distinguishing maternal, paternal and sibling effects has some potential to provide insight into the role of assortative mating and population stratification, the effects of which would impact estimates of the indirect effects of first‐degree relatives equally. Where three generations of genomic data are available, it will be possible to estimate parental genetic nurture effects free of bias from population stratification and assortative mating.

Some have highlighted that genetic nurture effects are akin to shared environmental effects in twin models, in that they are environmental effects that increase twin/sibling similarity (Cheesman, Eilertsen, et al., 2020; De Zeeuw et al., 2020). One study, in which twin models and intergenerational SNP heritability models were run on the same data set, reported that the non/significance of genetic nurture effects mirrored that of the shared environment (significant for child depression, not for anxiety; Cheesman, Eilertsen, et al., 2020). This raises a question: ‘are studies of genetic nurture simply reiterating what we have learnt about the shared environment from twin studies, or is there reason to think they will teach us something new?’ Several points are worth considering: First, genetic nurture is only one component of the shared environment, which also captures influences not indexed by the parental genome. Second, in most twin models, it is not possible to estimate covariance between the shared environment and genetic effects (although some extended family models allow for this, e.g. Keller et al., 2009), and where such covariance exists, this biases heritability and shared environment estimates (Lynch & Walsh, 1998; Rijsdijk & Sham, 2002). SNP heritability approaches to estimating genetic nurture do however allow for the estimation of covariance between indirect parental genetic effects and direct genetic effects (Eaves et al., 2014; Eilertsen et al., 2021; Young et al., 2018), so such methods may lead to insight not possible using twin methods. Third, shared environment estimates are variance components indicating the proportion of trait variance in a sample attributable to environmental effects shared by twins. They do not indicate which twins are exposed to high/low levels of shared environmental risk. However, when genetic nurture is estimated using parental PGS, we have an indicator of each individual's level of exposure. As such, parental PGS has more potential to teach us about within‐family influences than shared environmental estimates. They are flexible and can be used in causal inference designs (Balbona et al., 2021; Kim et al., 2020; Lawlor et al., 2017) and as potential moderators of child direct genetic effects and/or other predictors of child outcome.

### What can we expect family‐based genomic methods to teach us about psychopathology?

Multiple methods applied to multiple data sets have convincingly demonstrated that estimates of genetic influence on educational attainment are inflated, with studies indicating that genetic nurture effects are about half the size of direct genetic effects (Bates et al., 2018; Cheesman, Hunjan, et al., 2020; De Zeeuw et al., 2020; Kong et al., 2018; Selzam et al., 2019; Young et al., 2018). But what about psychopathology? At the time of writing, the evidence base is considerably smaller for mental health than for education, and it is not yet clear how important genetic nurture (and associated phenomena) is for mental health phenotypes. Several studies have reported no evidence for genetic nurture in psychopathology: An adoption study reported no evidence for genetic nurture affecting associations between psychiatric PGSs and body composition traits (Hunjan et al., 2021). Another study reported no link between non‐transmitted ADHD or educational attainment PGSs and child ADHD (De Zeeuw et al., 2020). And no evidence for genetic nurture was found using PGSs to predict neuroticism, schizophrenia or ADHD in a twin difference study (Selzam et al., 2019). Other studies have found evidence for genetic nurture: An SNP heritability study reported that indirect parental genetic effects explained 14% of the variance in child depressive symptoms at age 8 (child genetic effects explaining 19%; Cheesman, Eilertsen, et al., 2020). This finding was validated in a within‐sibship GWAS of depressive symptoms, in which effect sizes were attenuated by 39% (Howe et al., 2021). In another study, it was found that maternal PGSs for neuroticism and autism spectrum disorder predicted child ADHD symptoms (β = .05 in each case; Pingault et al., 2021), although many other maternal and paternal PGSs did not.

In attempting to interpret the few family‐based genomic studies to have examined psychopathology, it is important to consider that GWAS sample sizes for psychiatric outcomes are considerably smaller than for educational attainment, leading to PGSs that explain less genetic variance. As GWAS sample sizes increase over the coming years, the increased power may lead to more evidence for genetic nurture in mental health phenotypes, as with educational attainment. Those studies reporting significant indirect parental genetic effects have tended to be much larger than those reporting none, so this does seem reasonable. On the other hand, there are reasons to believe that effects may be greater for educational attainment than for mental health outcomes. For example, twin studies show that shared environmental effects for educational attainment are significant and persist into adulthood (Silventoinen et al., 2020). This is not the case for most human traits: shared environment explains some variance in emotional and behavioural problems in childhood but little to none by early adulthood (Hannigan, Walaker, Waszczuk, McAdams, & Eley, 2017). If genetic nurture is capturing some of the same variance indexed by the shared environment, then we should perhaps not expect the effects of genetic nurture to be large for traits with low shared environment estimates.

Whether or not genetic nurture estimates and associated phenomena such as assortative mating prove to be as large for mental health as for educational attainment, family‐based genetic research will play an important role in the study of psychopathology over the coming years. The methods outlined in this review will help researchers to clarify the role that parents play in the development of mental health problems, the ways in which children influence parent behaviour and how these processes impact estimates of genetic influence. We anticipate that researchers will make use of longitudinal databases to examine developmental change in genetic nurture and associated effects and to explore the longevity of any parental effects detected. We also expect investigations that explore interactions between direct and indirect genetic effects, as well as their moderation by factors such as child and parent sex, environmental context and age.

### Quasi‐experimental family methods not covered

We did not cover the classical twin design in this review, although we did cover extended twin/family studies and the use of twins in sibling comparison studies. As with the other methods discussed, researchers have combined the classical twin design with genomic data (Dolan, Huijskens, Minică, Neale, & Boomsma, 2021; Minică, Dolan, Boomsma, De Geus, & Neale, 2018), and this is likely to be an exciting area of methodological development in the future. There are also several quasi‐experimental designs that make use of stepfamilies, parents who do not live with their children and half‐siblings (Kendler, Ohlsson, Sundquist, & Sundquist, 2015, 2019, 2020a, 2020b). Where genomic data are available on such families, this could provide an interesting avenue for future research.

We did not cover intergenerational Mendelian randomisation, a method wherein parental variants/PGSs are used as instrumental variables to determine the causal influence of a parent trait on a child trait. Using non‐transmitted parental alleles strengthens this method (Lawlor et al., 2017). Indeed, some of the first studies to make use of the non/transmitted allele distinction come from the Mendelian randomisation literature (Zhang et al., 2015). Intergenerational Mendelian randomisation has not yet been used in the study of psychopathology, but likely will in the future, and forms a key component of some of the methods we have discussed (Balbona et al., 2021; Kim et al., 2020).

### Limitations, considerations and challenges

We have shown that family‐based quasi‐experimental methods can be used to address some limitations of genomic research, and vice versa, genomic data can improve quasi‐experimental family research. That said, limitations remain. A major shortcoming is that most genomic and family databases comprise socioeconomically privileged participants from Western, educated, industrialised, rich, democratic (WEIRD) countries, predominantly in Europe and the USA. These samples tend to be culturally homogeneous, comprising participants of European heritage. To what extent findings derived from such data can be generalised to other populations within WEIRD countries, or to other populations across the globe, is an open question that can only be answered by collecting more data on more diverse samples. There are reasons to suppose that findings may not generalise beyond WEIRD samples. For example, cross‐cultural twin studies report differences in aetiology for mental health (Ball et al., 2009, 2011; Zavos et al., 2020). At present, the focus on WEIRD populations means that our scientific knowledgebase is weighted towards serving privileged groups and limits the transferrable, clinical utility of results.

Most current genomic methods only capture a portion of genetic influences captured by twin and adoption studies because effects tagged by common SNPs do not capture a trait's full genetic variance (see glossary definition of SNP heritability for more details). As such, estimates of direct and indirect genetic effects calculated using the methods covered in this review will be downwardly biased. Furthermore, PGSs are limited by the GWAS summary data used to generate them. For example, where PGSs are generated using GWAS results derived from WEIRD databases, they explain less variance if used in non‐WEIRD samples (Duncan et al., 2019). Most GWAS of mental health phenotypes focus on adult diagnoses, so where family studies include children, it is worth considering to what extent genetic risk for adult mental health problems should be expected to overlap with mental health issues in children. While there is evidence to suggest that PGSs for adult diagnoses predict emotional and behavioural difficulties earlier in life (Akingbuwa et al., 2020), there is also evidence to suggest that child and adult mental health problems can be genetically distinct (Ask et al., 2021; Eley et al., 2015).

An important part of any scientific endeavour is the replication of findings and triangulation across studies using distinct methods. At present this is a challenge when using genetically informative family databases because so few samples exist. This can make it impossible to distinguish the effects of using a specific research design versus a specific sample (Ahmadzadeh et al., 2021). One advantage of collecting genomic data on the participants of large family‐based databases is that results obtained using distinct research designs can be compared within a sample: For example, SNP and pedigree heritability (Cheesman, Eilertsen, et al., 2020; Jami et al., 2020), twin‐based and PGS effects (Selzam et al., 2018), or adoption and sibling PGS designs (Demange et al., 2022).

A major reason for there being so few family‐based genetic databases is that they are logistically challenging and expensive to create. To circumvent some of these challenges, and to make best use of available resources, several genomic data sets are being extended to recruit the family members of participants. This provides an economical way to create family databases. However, by recruiting from the families of study participants it is possible that selection biases (Munafò, Tilling, Taylor, Evans, & Davey Smith, 2018; Pirastu et al., 2021; Taylor et al., 2018; Tyrrell et al., 2021) will be magnified and become greater than they would in a family sample drawn from the general population.

One possible solution to the lack of family‐based genomic databases (besides collecting more data) is to impute the genomes of missing family members: when data are available on siblings, it is possible to (partially) recreate parent genomes and thus to estimate genetic nurture (Hwang et al., 2020; Young et al., 2022). This solution presents a means to extend current genetic databases while avoiding the need for further recruitment or data collection, although does not help to address the existing lack of diversity among participating families.

## Summary

In this review, we have shown how family‐based quasi‐experimental research designs are being combined with genomic data and how doing so is providing researchers with an increasingly sophisticated toolbox to use in disentangling the role of genetic and environmental effects in human psychiatric traits. This is an exciting time for research. Increasingly, distinct fields are converging and borrowing methodological approaches from one another. Clearly, genetic research can contribute an enormous amount of insight to any study of human populations, and the increasing availability of genomic data brings with it many exciting possibilities. We have discussed how DNA can provide new means to study the ways in which family members influence one another's mental health and how this is likely to affect our understanding of psychopathology over the coming years. We have also highlighted that it is important to consider that genotype–phenotype associations are much like any other association – they may be causal in nature, but they may also be spurious, inflated, suppressed or partially or entirely confounded. Given this, the use of quasi‐experimental methods to try to distinguish direct, causal genetic effects from non‐causal associations is an extremely important step on the road to better understanding aetiology. It is highly likely that the approaches we have summarised in this review are only the beginning – there will be further methodological developments, new data collected and continued scientific creativity. We have highlighted some of the ways in which we think the merging of family‐based quasi‐experimental methodology with genomic data will inform our understanding of psychopathology over the coming years. We look forward to new developments yet to come.

## Supporting information


**Appendix S1.** Glossary.

## Endnotes

^1^
Confounding effects not shared by siblings will not be accounted for in sibling comparison studies unless explicitly assessed and controlled for (Frisell, Öberg, Kuja‐Halkola, & Sjölander, 2012).

^2^
Although it is *possible* that this assumption will not hold for some antenatal exposures. E.g. foetal genetic factors could feasibly influence maternal stress response in such a way that partially explains differences in maternal emotions or behaviour between pregnancies.

^3^
When possible, it is important to jointly model the effects of maternal and paternal genotype on child phenotype. Modelling the effect of a single parent genotype on a child phenotype controlling for child genotype can induce a negative covariance between paternal and maternal genotype (via collider bias), and thus downwardly bias genetic nurture estimates (Lawlor et al., 2017).
