# Supplementary material for: Annual Research Review: Towards a deeper understanding of nature and nurture: combining family‐based quasi‐experimental methods with genomic data
Source: J Child Psychol Psychiatry. 2022 Nov 15;64(4):693–707. doi: 10.1111/jcpp.13720 (PMC10952916; doi:10.1111/jcpp.13720)
Supplement: Supplementary file 1 — Appendix S1. Glossary. [file JCPP-64-693-s001.pdf]

Glossary

Alleles are versions of genetic variants. At every locus on the genome, humans possess two alleles, one inherited from their mother, and one from their father. When a human creates gametes (sex cells), each gamete receives one of those alleles. This process is random, each gamete having a 50% chance of receiving either allele. In this manner, genetic differences between siblings are entirely random and so independent of passive gene-environment correlation, indirect genetic effects, assortative mating, or population stratification.

Assortative mating describes the tendency for people to mate non-randomly, typically by choosing partners who are similar to themselves on particular characteristics. Many studies show that partners correlate with one another on a wide range of traits including mental health diagnoses (Nordsletten et al., 2016; Peyrot, Robinson, Penninx, & Wray, 2016). Assortative mating is likely to—at least partially—explain these similarities[1]. When parents assort on a heritable trait then their children are likely to inherit a more extreme genetic load for that trait. Where exposure to parent trait plays a role in the development of child trait, children will also experience a more extreme 'environmental load' because both parents will be similar in that trait. In this manner assortative mating can increase any existing passive gene-environment covariance (Keller et al., 2009).

Genome-wide association studies (GWAS) comprise a hypothesis-free approach to testing for associations between a trait and millions of single nucleotide polymorphisms (SNPs) across the genome (Pearson, 2008; Visscher et al., 2017). SNPs are by far the most common form of genetic variant (way in which human DNA differs between people) and as such explain a majority of genetic variation in human populations. Where GWAS identify significant associations between SNPs and a trait, and where these associations are causal, they have the potential to provide insight into the biological processes underlying development of that trait. Because effect sizes for most SNPs are extremely small, and because GWAS requires correction for potentially millions of tests, a well-powered GWAS requires a very large sample size. For example, the largest GWAS thus far conducted involved 1.1 million individuals (Lee et al., 2018). The largest mental health GWAS involved ~70,000 people with schizophrenia and ~237,000 controls (Ripke, Walters, & O'Donovan, 2020). Large GWASs have now been conducted for many traits of interest to mental health researchers.

Polygenic scores (PGS) are quantitative scores that indicate genetic liability for a trait (Choi, Mak, & O'Reilly, 2020). PGSs sum the weighted effects of multiple genetic variants identified in a GWAS as associated with a trait. In this manner summary data from GWASs (constituting effect sizes and significance of SNPs) can be used to construct PGSs for an extremely broad range of traits for any individual who has had their genome sequenced. Although GWAS require very large samples to detect the small effect sizes of individual SNPs, samples of only a few hundred individuals can provide power to detect associations between PGS and traits (dependent on effect size).

[1] Other phenomena could also play a role such as phenotypic convergence whereby partners socially influence one another and become more similar over time, or social homogamy, whereby partner similarities are attributable to shared social strata. NB elsewhere, phenotypic convergence and social homogamy have been referred to as *types* of assortative mating. Our definition of assortative mating aligns with what has been referred to as *primary assortative mating*.

Population stratification describes the situation in which allele frequencies differ between subpopulations in a population (Cardon & Palmer, 2003). It is a product of non-random mating within a population across many generations. Often these mating patterns are driven by geographic and/or sociocultural factors (i.e. factors determining proximity and interaction) which, when combined with random genetic differences and genetic drift (changes in allele frequencies from generation to generation attributable to random deaths and variation in the numbers of children born) lead to the formation of genetically distinct subpopulations. Where these subpopulations also differ on a phenotype (perhaps because of the same geographic and sociocultural factors that impacted mating patterns), then population stratification can lead to spurious genotype-phenotype associations. For example, where population stratification has led to genetic differences between subpopulations who also differ in terms of their diet, then a naive analysis that combines those subpopulations without correcting for population stratification could erroneously infer associations between those genetic differences and traits linked with dietary differences (e.g., many health-related traits). Population stratification is a source of passive gene-environment correlation.

SNP heritability is a measure of heritability that can be defined as the proportion of trait variance in a population explained by measured SNP variance in that population (i.e., the extent to which differences in Single Nucleotide Polymorphisms in a population can explain differences in a given trait in that population) (Yang, Zeng, Goddard, Wray, & Visscher, 2017). SNP heritability estimates tend to be lower than heritability estimates derived from twin or family studies because SNP heritability captures only the additive effects of common SNPs. Heritability estimates from twin and family studies include the effects of all genetic variation. This will include non-additive genetic variation (when not explicitly modelled), as well as the effects of rare variants not indexed by common SNPs.

References

Cardon, L. R., & Palmer, L. J. (2003). Population stratification and spurious allelic association. *The Lancet, 361*(9357), 598-604. doi:10.1016/s0140-6736(03)12520-2

Choi, S. W., Mak, T. S.-H., & O'Reilly, P. F. (2020). Tutorial: a guide to performing polygenic risk score analyses. *Nature Protocols, 15*(9), 2759-2772. doi:10.1038/s41596-020-0353-1

Keller, M. C., Medland, S. E., Duncan, L. E., Hatemi, P. K., Neale, M. C., Maes, H. H. M., & Eaves, L. J. (2009). Modeling Extended Twin Family Data I: Description of the Cascade Model. *Twin Research and Human Genetics, 12*(1), 8-18. doi:10.1375/twin.12.1.8

Lee, J. J., Wedow, R., Okbay, A., Kong, E., Maghzian, O., Zacher, M., . . . Cesarini, D. (2018). Gene discovery and polygenic prediction from a genome-wide association study of educational attainment in 1.1 million individuals. *Nature Genetics, 50*(8), 1112-1121. doi:10.1038/s41588-018-0147-3

Nordsletten, A. E., Larsson, H., Crowley, J. J., Almqvist, C., Lichtenstein, P., & Mataix-Cols, D. (2016). Patterns of Nonrandom Mating Within and Across 11 Major Psychiatric Disorders. *JAMA Psychiatry, 73*(4), 354. doi:10.1001/jamapsychiatry.2015.3192

Pearson, T. A. (2008). How to Interpret a Genome-wide Association Study. *JAMA, 299*(11), 1335. doi:10.1001/jama.299.11.1335

Peyrot, W. J., Robinson, M. R., Penninx, B. W. J. H., & Wray, N. R. (2016). Exploring Boundaries for the Genetic Consequences of Assortative Mating for Psychiatric Traits. *73*(11), 1189. doi:10.1001/jamapsychiatry.2016.2566

Ripke, S., Walters, J. T., & O'Donovan, M. C. (2020). *Mapping genomic loci prioritises genes and implicates synaptic biology in schizophrenia*. Cold Spring Harbor Laboratory. Retrieved from https://dx.doi.org/10.1101/2020.09.12.20192922

Visscher, P. M., Wray, N. R., Zhang, Q., Sklar, P., McCarthy, M. I., Brown, M. A., & Yang, J. (2017). 10 Years of GWAS Discovery: Biology, Function, and Translation. *The American Journal of Human Genetics, 101*(1), 5-22. doi:10.1016/j.ajhg.2017.06.005

Yang, J., Zeng, J., Goddard, M. E., Wray, N. R., & Visscher, P. M. (2017). Concepts, estimation and interpretation of SNP-based heritability. *Nature Genetics, 49*(9), 1304-1310. doi:10.1038/ng.3941
